# Intelligence

**Published:** 1810-01

**Authors:** 


### INTELLIGENCE.

A very curious fœtus has lately been brought into the world; all the parts of which, from the head to the upper part of the abdominal viscera were double; what is still more remarkable, the organs of generation were double, complete, and exhibited separately the parts of each sex, external and internal. The vast and inconvenient volume of the two heads, rendered it impossible to bring them alive into the world.

Common spirits of turpentine have been recently administered by several medical gentlemen of the metropolis, with good effect, in the cure of tape worm. The doses given were in some cases so large as two ounces, but those of half an ounce at a time, repeated twice a day, were generally found to answer the purpose. The vehicle in which the turpentine was administered, was generally honey.

Doctor VAN MARUM has discovered a very simple method, proved by repeated experiments, of preserving the air pure in large halls, theatres, hospitals, &c. The apparatus for this purpose is nothing but a common lamp, made according to Argand's construction, suspended from the roof of the hall, and kept burning under a funnel, the tube of which rises above the roof without, and it is furnished with a ventilator. For his first experiment, he filled his large laboratory with the smoke of shavings. A few minutes after he lighted his lamp, the whole of the smoke had disappeared, and the air was perfectly purified.

M. CHAPTAL has recently made experiments so as to ascertain the nature of seven specimens of colours, found in a colour-shop at Pompeji. No. 1, the only one which has not received any preparation from the hand of man is a greenish and saponaceous argil, in the state in which Nature presents it in various parts of the globe, and resembling that known by the name of the Terra di Verona. No. 2, is an arch of a beautiful yellow, all the impurities of which have been removed by washing. As this substance turns red by calcination with a gentle fire, the yellow colour, which it has preserved without alteration, affords a new proof, that the ashes which covered Pompeji retained but a slight degree of heat. No. 3, is a brown red, like that employed at present for coarse work, and is produced by the calcination of the preceding. No. 4, is a pumice stone, extremely light and white; the texture is very fine and close; the three others are compound colours, which M. Chaptal was obliged to analyse, in order to ascertain the constituent principles. From his experiments on No. 5, which is of a deep blue, and in small pieces of the same form, it appears to be composed of oxyd of copper, lime, and alumine. It resembles ash blue in the nature of its principles, but differs from them in its chemical properties. It seems to be the result, not of precipitation, but of the commencement of vitrification; and the process, by which which it was obtained by the ancients, is lost. No. 6, is a sand of a light blue, mixed with some small whitish grains. On analysing it, M. Chaptal discovered in it the same principles as in the preceding; indeed, it may be considered as a composition of the same nature, in which there is a greater proportion of lime and alumine. No. 7, is of a beautiful roseate hue; it is soft to the touch; is reduced between the fingers to an impalpable powder: and leaves upon the skin a pleasing carnation colour. From M. Chaptal's experiments, he looks upon it as a real lake, in which the colouring principle is united with alumine. In its properties, its hue, and the nature of its colouring principle, it has nearly a complete analogy with madder lake. The preservation of this lake for nineteen centuries, without perceptible alteration, is a phenomenon which cannot fail to excite the astonishment of chemists.

For some time, the curiosity of the Parisians has been gratified, by Messrs. Franconi, with a spectacle truly extraordinary, that of the most shy and timid animal, a stag, tamed and trained to the same performances as the most docile and courageous horse. Led by his instructor, the docile animal advances into the arena, looking round on every side, with an air equally expressive of gentleness and intelligence. At the command of his master he bends his knees, and respectfully bows his head. M. Franconi mounts his back, cracks his whip, and fires pistols, at which the animal shews neither fear nor alarm. After this first experiment he is left to himself, and made to perform the exercises of the manege, like the best-trained horse. He sets off at full gallop; turns and stops at the word of command. He leaps over rails with wonderful agility, and even clears two horses at once. After every performance he stands still, fixes his eyes on his master, and endeavours to discover from his looks whether he is satisfied. Mr. Franconi then goes up to him, pats him, and bestows other caresses, for which the gentle animal testifies the highest gratitude. In the last place, a triumphal arch, charged with fire-woiks, is erected in the middle of the air; it is set on fire, and the stag, impatient for the signal, starts off, as soon as it is given, and passes twice under the blazing arch, amidst the shouts and applauses of the spectators.

We are disappointed in our expectations of the account of the man bitten by the rattle-snake. A very ingenious paper on the case, by Mr. Home, has, however, been read before the Royal Society, giving an account also of other snakes in America, and of the cobre de cabello in Asia. We shall, in a future number, offer the substance of this communication.

We have received from Dr. Andrews, and the other physicians of Madeira, English and Portuguese, an accurate account of the success of vaccination in that island. This account is chiefly intended to answer the unfavourable representations of Dr. Carneiro. As it contains also some valuable information relative to the epidemic measles, hooping-cough, and dysentery, introduced by the military, we shall give the whole in our next.

Dr.

Dr. BUXTON's Spring Course of Lectures, on the Theory and Practice of Medicine, will be commenced about the middle of January, 1810, at the Medical Theatre, London Hospital.

Dr. CLARKE and Mr. CLARKE will begin their Spring Course of Lectures on Midwifery and the Diseases of Women and Children, on Monday January the 29th, 1810. The Lectures are read every day, at the house of Mr. Clarke, No. 10, Upper John Street, Golden Square, from a quarter past Ten o'clock in the morning 'till a quarter past Eleven, for the convenience of Students attending the Hospitals. For particulars, apply to Dr. Clarke, No. 1, New Burlington Street; or to Mr. Clarke, No. 10, Upper John Street, Golden Square.

Dr. CLUTTERBUCK will begin his Spring Courses of Lectures on the Theory and Practice of Physic, Materia Medica, and Chemistry, on Monday, January the 22d, at a Quarter before Ten in the Morning, at his House, No. 1, Crescent, New Bridge Street, Blackfriars; where further Particulars may be had.

Dr. RAMSBOTHAM will read the Introductory Lecture to his next general Course on the Science and Practice of Midwifery, on Wednesday January the 3d, 1810, at Seven o'clock in the Evening, at his house, No. 9, Old Jewry.

Dr. REID's next Course of Lectures on the Theory and Practice of Medicine, will commence on Wednesday, the 3d of January. The Introductory Lecture will be delivered on that day, at six o'clock in the evening; and the subsequent Lectures will be given at the same hours, on Mondays, Wednesdays, and Fridays, until the conclusion of the Course on Friday, the 23d of March.

Dr. SQUIRE will begin a Course of Lectures on Midwifery, and the Diseases of Women and Children, on Tuesday January 16.—For particulars apply to Dr. SQUIRE, 30, Ely Place, Holborn.

Mr. TAUNTON's Spring Course of Lectures on Anatomy, Physiology, Pathology, and Surgery, will commence on Saturday January the 27th, 1810, at Eight o'clock in the Evening *precisely*, and be continued every Tuesday, Thursday, and Saturday, at the same hour. Particulars may be had on applying to Mr. Taunton, Greville-Street, Hatton Garden.

---

Dr. ADAMS has published his new edition of Mr. Hunter on the Venereal Disease. The Text is unaltered; the Introduction and Commentaries are very copious. The whole is included in a single 8vo volume.

Dr. TROTTER will shortly publish "An Appeal to the Officers of the Royal Navy, on the Measures which have been resorted to, to deprive him of the increased Pay lately granted to Naval Physicians. With official Documents, Certificates of Officers, and Reflections on the present Medical Establishment."

Dr. UWINS, of Aylesbury, has in the Press, just ready for Publication, a small Tract, entitled, Cursory Remarks on the Causes,

Causes, Prevention, and Treatment of Fever, occasioned by the recent occurrence of an Epidemic Disorder in Aylesbury and its Neighbourhood.

---

### *Diseases and Casualties in London in the Year* 1809.

| | | | | | |
|---|---|---|---|---|---|
| Abortive and Stillborn | 51 | Grief | 5 | Water in the Cheſt | 11 |
| Abſceſs | 49 | Jaundice | 26 | Water in the Head | 252 |
| Aged | 1251 | Jaw Locked | 4 | Worms | 5 |
| Ague | 4 | Inflammation | 511 | | |
| Apoplexy & Suddenly | 203 | Influenza | 3 | | |
| Aſthma and Phthiſic | 488 | Livergrown | 21 | | |
| Bile | 2 | Lunatick | 166 | Bit by a Rattleſnake | 1 |
| Bleeding | 24 | Meaſles | 106 | Bit by a Mad Dog | 1 |
| Burſten and Rupture | 15 | Miſcarriage | 2 | Bruiſed | 5 |
| Cancer | 55 | Mortification | 167 | Burnt | 30 |
| Childbed | 123 | Palſy | 123 | Drowned | 124 |
| Colds | 15 | Palpitation of the Heart | 1 | Exceſſive Drinking | 7 |
| Colic, Gripes, &c. | 15 | Pleuriſy | 19 | Executed | 6 |
| Conſumption | 4570 | Quinſy | 3 | Found Dead | 8 |
| Convulſions | 3463 | Rheumatiſm | 2 | Fractured | 2 |
| Cough, and Hooping Cough | 591 | Scarlatina | 1 | Frighted | 1 |
| | | Scurvy | 4 | Frozen | 1 |
| Cramp | 2 | Small Pox | 1163 | Killed by Falls and ſeveral other Accidents | 68 |
| Croup | 81 | Sore Throat | 7 | | |
| Diabetes | 1 | Sores and Ulcers | 5 | Killed themſelves | 52 |
| Dropſy | 736 | Spaſm | 24 | Murdered | 1 |
| Evil | 2 | St. Anthony's Fire | 2 | Overjoy | 1 |
| Fevers of all kinds | 1066 | St. Vitus's Dance | 1 | Poiſoned | 4 |
| Fiſtula | 3 | Stoppage in the Stom. | 20 | Scalded | 5 |
| Flux | 9 | Strangury | 1 | Smothered | 1 |
| French Pox | 29 | Teeth | 308 | Starved | 1 |
| Gout | 30 | Thruſh | 39 | Suffocated | 7 |
| Gravel, Stone, and Strangury | 10 | Tumour | 1 | Total | 326 |

| | | | |
|---|---|---|---|
| Chriſtened | Males 9981 | Females 9631 | In all 19612 |
| Buried | Males 8636 | Females 8044 | In all 16680 |

Whereof have died,

| | | | |
|---|---|---|---|
| Under Two Years of Age | 4937 | Fifty and Sixty | 1419 |
| Between Two and Five | 1916 | Sixty and Seventy | 1235 |
| Five and Ten | 754 | Seventy and Eighty | 1063 |
| Ten and Twenty | 506 | Eighty and Ninety | 369 |
| Twenty and Thirty | 1145 | Ninety and a Hundred | 54 |
| Thirty and Forty | 1472 | A Hundred | 2 |
| Forty and Fifty | 1748 | | |

Decreaſed in the Burials this Year 3274.

This year appears to have been particularly healthy, the decrease of deaths being 3274, compared with the preceding, which had not exceeded the year 1807 more than 1620. This decrease cannot be imputed to a diminished population of that part of the town which is included within the bills, because the number of christenings come within three hundred of the last year. Small-pox remains nearly the same as last year, which shows the disease has been mild, as it is notorious that it has been more general.

The

The mortality among young Children, taking them to the age of five, is less this year than the last by 1688. As usual, the greatest mortality of adults has been between 40 and 50, a space which comprehends the most dangerous climacteric among the labouring part of the community. The succeeding decade is next in fatality, comprehending a similar critical period with those in easier circumstances.

Though scarlet fever is known to have been prevalent and fatal, yet the article of fevers of all kinds is less by nearly a hundred than last year. How much is it to be regretted, that these records should be so imperfect in articles of such importance. The same may be said of hooping-cough, which has been so prevalent this year, but being included in coughs in general, we cannot ascertain its increase with any certainty. The whole number who have died of coughs is greater than last year by 173. Probably, the comparative increase of hooping cough has been much greater if we may estimate the other coughs by the general healthiness of the season, which last would have less effect on a contagious disease. The proportion of male births beyond female is about the usual average. The average excess of deaths in the males is somewhat more than usual. The only increase is in the articles of croup and cough.

Among the casualties, only one is reported as bitten by a mad dog. The others, whom recollection informs us are not few, we suppose were buried without the bills.

How strange that two parishes so well regulated as Mary-le-bone and St. Pancras, should furnish no registers! How interesting would an account of the gradual increase of each have proved from year to year!

